# Imaging the Abdominal Manifestations of Cystic Fibrosis

**DOI:** 10.1155/2017/5128760

**Published:** 2017-01-29

**Authors:** C. D. Gillespie, M. K. O'Reilly, G. N. Allen, S. McDermott, V. O. Chan, C. A. Ridge

**Affiliations:** ^1^Department of Medicine, Mater Misericordiae University Hospital, Dublin, Ireland; ^2^Department of Radiology, Mater Misericordiae University Hospital, Dublin, Ireland; ^3^University College of Dublin School of Medicine, Dublin, Ireland; ^4^Department of Radiology, Massachusetts General Hospital, 55 Fruit St., Boston, MA 02114, USA

## Abstract

Cystic fibrosis (CF) is a multisystem disease with a range of abdominal manifestations including those involving the liver, pancreas, and kidneys. Recent advances in management of the respiratory complications of the disease has led to a greater life expectancy in patients with CF. Subsequently, there is increasing focus on the impact of abdominal disease on quality of life and survival. Liver cirrhosis is the most important extrapulmonary cause of death in CF, yet significant challenges remain in the diagnosis of CF related liver disease. The capacity to predict those patients at risk of developing cirrhosis remains a significant challenge. We review representative abdominal imaging findings in patients with CF selected from the records of two academic health centres, with a view to increasing familiarity with the abdominal manifestations of the disease. We review their presentation and expected imaging findings, with a focus on the challenges facing diagnosis of the hepatic manifestations of the disease. An increased familiarity with these abdominal manifestations will facilitate timely diagnosis and management, which is paramount to further improving outcomes for patients with cystic fibrosis.

## 1. Introduction

Cystic fibrosis (CF) is the most common inherited fatal disease in Caucasians. Ireland has the highest birth incidence of the disease in the world, affecting 1 in every 1600 births [1]. Its inheritance pattern is autosomal recessive but can be a result of one of over 1500 mutations of the cystic fibrosis transmembrane regulator (CFTR) gene, located on the long arm of chromosome 7 [2, 3]. The most common of these is the deletion of phenylalanine at position 508: F508del.

The CFTR gene and its product, a cyclic adenosine monophosphate (cAMP) mediated chloride channel, play a key role in hydrating bodily secretions and regulating cellular functions including sodium transport [3]. The defective gene product causes impaired chloride ion transport across exocrine gland epithelial cells [4] which leads to thickened viscous secretions affecting multiple organs including the lungs, liver, and pancreas.

With improvements in the management of the respiratory complications of CF, life expectancy in this population has increased. The abdominal manifestations of CF are common and may not present until adulthood in some cases. Many challenges remain in the diagnosis of these abdominal manifestations, particularly in the area of cystic fibrosis-related liver disease (CFLD). CFLD is an independent risk factor for mortality in CF [5] and a significant cause of morbidity and mortality in a proportion of CF patients. A reliable noninvasive method for early prediction of significant CFLD or cirrhosis is lacking. Consequently, a familiarity with the available screening tools and their limitations, the expected imaging appearances of CF abdominal disease, and the areas of research likely to impact on their diagnosis in future is paramount.

Adults comprise 45% of patients with CF in Ireland [1]. We selected the most representative abdominal imaging findings in patients with CF across all imaging modalities performed in two academic health centres. In this pictorial review, we present the typical abdominal ultrasound (US), computed tomography (CT), and magnetic resonance imaging (MRI) findings in adults with CF, with a focus on CF related liver disease.

The aim of this review is to heighten awareness of the abdominal manifestations of CF and their imaging findings, such that clinicians are better equipped to provide a timely diagnosis for what is an increasingly significant range of conditions in this population.

## 2. Pancreatic Manifestations

The pancreas is the most commonly affected abdominal organ in CF. The pathophysiology, clinical manifestations, and imaging findings of the typical pancreatic manifestations will be discussed.

### 2.1. Pathophysiology and Clinical Manifestations

Pancreatic disease is caused by inspissation of abnormally concentrated pancreatic secretions which produces proximal duct obstruction. This results in acinar atrophy, inflammation, progressive fibrosis, fatty replacement, calcification, and cyst formation.

Clinical manifestations of pancreatic dysfunction in CF include pancreatic insufficiency, pancreatitis, and an increased risk of pancreatic cancer compared to the general population [6]. Pancreatic insufficiency encompasses both exocrine and endocrine insufficiency. Exocrine occurs in 85% while endocrine occurs in 30–50% of CF patients [7]. It can lead to malabsorption and therefore malnutrition, poor health, and growth. It presents in childhood for the majority of patients, although patients who are diagnosed with CF in adulthood are less likely to have pancreatic insufficiency [7]. Pancreatitis is rare despite pathologic evidence of a chronic inflammatory process and is more common in pancreatic sufficient patients [7, 8].

### 2.2. Pathological and Imaging Findings

#### 2.2.1. Pancreatic Atrophy and Steatosis

**Figure 1:**
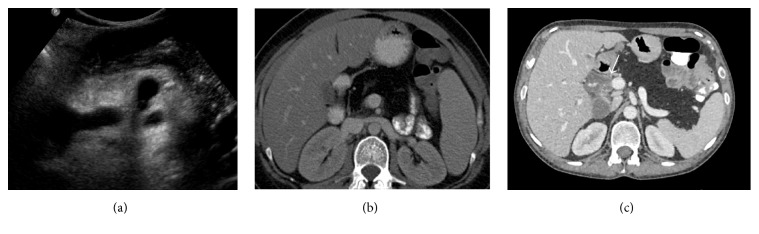
(a) Transverse abdominal ultrasound of a 34-year-old male with cystic fibrosis. Fatty infiltration of the pancreas is typically echogenic on US. (b) The pancreas is homogenously low in attenuation on CT and is often difficult to differentiate from normal retroperitoneal fat. (c) Axial portal venous phase contrast-enhanced CT performed to assess abdominal pain in a 44-year-old CF patient 2 weeks after bilateral double lung transplant demonstrates diffuse pancreatic lipomatosis which manifests as homogenous low attenuation. Note is also made of a distended common bile duct (white arrow) due to stricture.

The pancreas is commonly atrophic in CF and may have a pattern of atrophy with partial fatty replacement or atrophy with complete fatty infiltration. Fatty infiltration of the pancreas is typically echogenic on US (Figure 1(a)) and homogenously low in attenuation on CT, often difficult to differentiate from normal retroperitoneal fat (Figure 1(b)). On T1 weighted MRI, there is increased signal intensity representing fatty infiltration interspersed with low signal intensity representing fibrosis. The pancreas may be enlarged and replaced with fat, known as lipomatous pseudohypertrophy [9].

**Figure 2:**
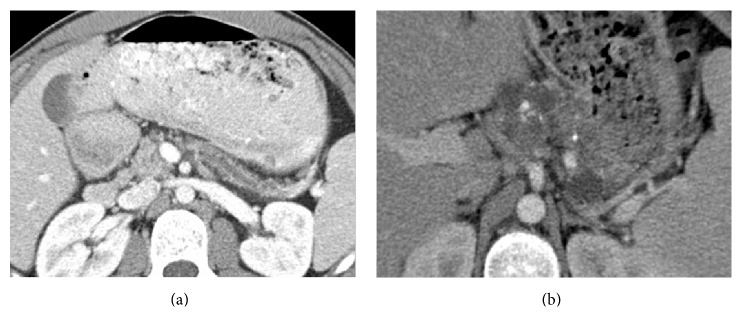
(a) Axial CT images of a 38-year-old male with cystic fibrosis. There is atrophy of the pancreas and duct irregularity. (b) Axial CT image of a 36-year-old female exhibiting pancreatic cysts, pancreatic calcification, and duct irregularity.

The atrophic pancreas with partial fatty replacement is small in size and echogenic on US. The CT features include loss of the normal soft tissue component of the gland which may be accompanied by calcification and duct irregularity (Figures 2(a) and 2(b)).

#### 2.2.2. Pancreatic Cystosis

Replacement of the pancreas with macroscopic cysts is rare. It is most likely a result of protein hyperconcentration, thickened secretions, and ductal ectasia (Figure 2(b)). The cysts are lined by epithelium and thus are true cysts. They replace normal pancreatic parenchyma, may displace but should not infiltrate adjacent structures, and do not exceed 5 cm in diameter as seen on US and CT (Figure 2(b)).

## 3. Renal Manifestations

Although renal complications in CF are infrequent, the most common manifestations include nephrolithiasis, electrolyte abnormalities, and acute kidney injury (AKI). Rarer manifestations include progression to chronic kidney disease (CKD) and parenchymal disease including amyloidosis, diabetic nephropathy, nephrocalcinosis, diffuse and nodular glomerulosclerosis, and calcineurin inhibitor toxicity in lung transplant patients [10].

**Figure 3:**
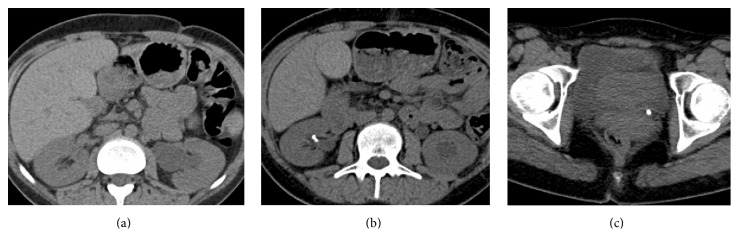
(a–c) Axial CT images of a 21-year-old female with left renal angle pain on a background of cystic fibrosis. (a) Noncontrast axial CT at the level of the renal hila demonstrates left hydronephrosis. The absence of normal pancreatic tissue and increased attenuation of the liver possibly due to haemosiderin deposition are also evident. (b) Axial CT image below the renal hila demonstrating a nonobstructing calculus in the right renal pelvis and left hydroureter. (c) Axial CT image at the level of the bladder demonstrating an obstructing left lower ureteric calculus.

Nephrolithiasis occurs in 3–6% of CF patients, three times more than age matched controls [11, 12]. It is the most common renal manifestation of CF (Figure 3). Urine is supersaturated with calcium oxalate, the main component of calculi in CF [13]. Hyperoxaluria is attributed to chronic antibiotic use, which reduces gastrointestinal colonisation by bacteria which normally degrade oxalate [12]. Oral fluids and a low oxalate diet are advised for prevention. Antegrade nephrostomy placement may be required in complicated cases.

CKD is rare, with an annual prevalence of 2.3% reported in one study of 12,000 CF adults. This doubled with every additional 10 years of age [14]. Recurrent episodes of AKI and aminoglycoside exposure are debated as the main contributors to CKD. Advanced CKD is rare, with a prevalence of 0.6% for stage 5 CKD and risk factors include lung transplant, CF related diabetes, and recurrent AKI [10].

## 4. Hepatobiliary Manifestations 

CFLD encompasses a broad spectrum of hepatobiliary complications including cholestasis, elevated aminotransferases, hepatic steatosis, hepatic fibrosis, focal biliary cirrhosis, and multilobular cirrhosis with or without portal hypertension. CF is also known to be complicated by microgallbladder and hepatocellular carcinoma (HCC) has been reported in four patients in the literature [6, 15–17].

### 4.1. Aetiology and Classification

CFLD is due to abnormally thickened secretions within the biliary epithelium, which then accumulates within bile ducts, concentrating caustic bile components in the liver [2]. Modifier genotypes in CF may also contribute to severity of CFLD. The z-allele of the SERPINA 1 gene has a strong association (odds ratio of 5) for developing CFLD with portal hypertension [18].

The fact that CFLD lacks a consistent definition is at least in part responsible for a significant variation in reported prevalence of CFLD in the literature. Studies using broader interpretations of the term report a prevalence of 30–40%, while focal biliary cirrhosis has been reported as occurring in 20–30% of CF individuals, with multilobular cirrhosis in 5–10% [19].

Liver cirrhosis is the most important extrapulmonary cause of death in CF, accounting for 2.5% of overall mortality [20], and is therefore the most clinically significant form of CFLD. The North American CF foundation proposed a classification for CFLD based on a consensus among hepatologists convened in 2007. This separates cirrhosis and portal hypertension from other forms of hepatobiliary involvement in CF (Table 1) [19].

### 4.2. Biochemical Abnormalities

A diagnosis of CFLD is considered if transaminases and gamma-glutamyl transferase (GGT) are above the upper limit of normal on at least three consecutive occasions within twelve months, with either hepatomegaly or splenomegaly on exam or ultrasound evidence of hepatobiliary abnormalities [20]. However, while basic liver function tests (LFTs) can be intermittently deranged in CF, they are largely nonspecific and are considered unreliable as a marker of severity or progression of CFLD. Meanwhile, patients with multilobular cirrhosis can have normal LFTs [20–22]. Research is ongoing to determine useful serum markers for early and reliable prediction of CFLD and its progression.

Studies have shown that different combinations of fibrogenesis markers such as collagen IV, prolyl hydroxylase, tissue inhibitor of metalloproteinase 1 levels, and monocyte chemoattractant protein 1 can distinguish patients with biopsy-proven CFLD from those with CF but no liver disease and early from late fibrosis [23, 24]. MicroRNAs also show promise, with studies showing various miRNAs can predict rapid onset of liver disease while specific combinations can distinguish fibrosis F0 from F3-4 [25]. These require further study for clinical implementation and are likely costly. Simpler biomarkers which are more readily available such as aspartate aminotransferase to platelet ratio (APRI) and Fib-4 have shown promise in one biopsy-controlled study; APRI differentiated CFLD from CF with no liver disease [sensitivity of 73%, specificity of 70%, in full agreement with histology staging 37% of the time and within one stage 73% of the time] while Fib-4 was shown to predict portal hypertension at diagnosis [25].

### 4.3. Biliary Manifestations

The biliary system can be involved in patients with CF but symptoms are rare. Inspissated biliary secretions cause mucosal hyperplasia in the gallbladder and bile ducts [26]. This process leads to increased sludge in the biliary tract and the development of microgallbladder, possibly through stenosis of cystic ducts and secondary atrophy of the gallbladder [6, 27].

**Figure 4:**
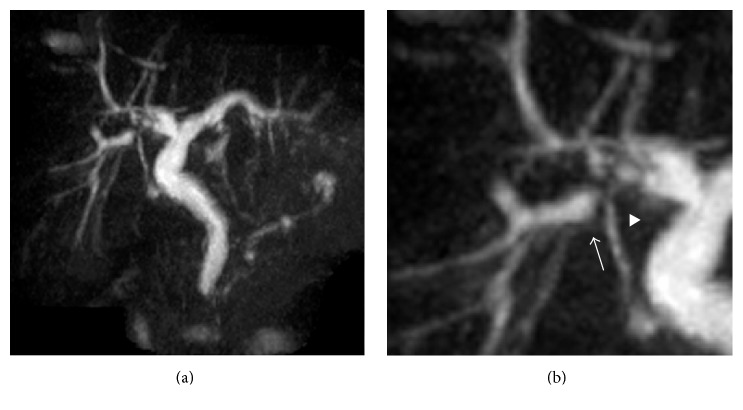
(a) Coronal half axis single shot turbo spin echo (HASTE) magnetic resonance cholangiopancreatography (MRCP) demonstrating intrahepatic biliary strictures in an asymptomatic 19-year-old female with CF. US performed to assess obstructive pattern abnormal liver function tests suggested biliary dilatation prompting MRCP. (b) Magnified MRCP image demonstrating focal biliary dilatation (white arrow) and biliary stricture (arrowhead).

**Figure 5:**
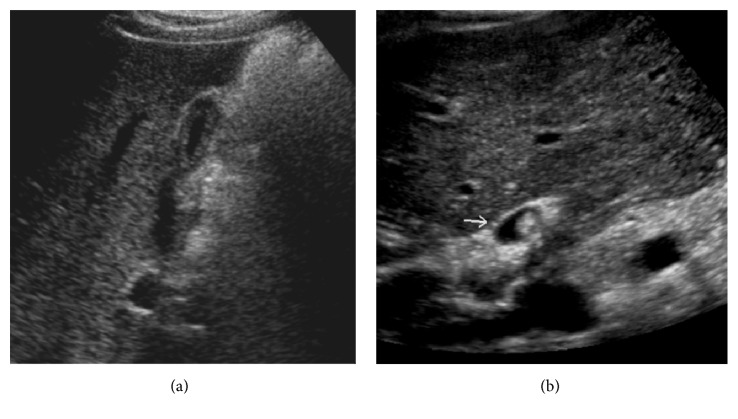
(a) US image of a 36-year-old female with cystic fibrosis and prior history of biliary colic demonstrating a persistently collapsed gallbladder (microgallbladder) in the longitudinal plane. (b) Gallstone in a contracted gallbladder (arrow).

**Figure 6:**
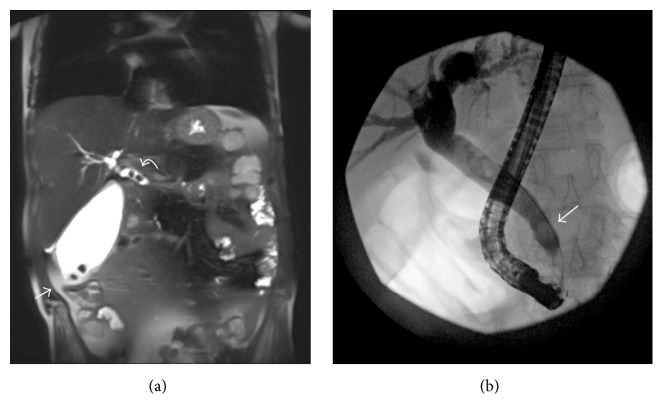
(a) Coronal T2 HASTE image of an MRCP performed to assess abdominal pain in a 44-year-old CF patient 2 weeks after bilateral double lung transplant demonstrates calculi in the distended fundus of the gallbladder. There is small volume pericholecystic fluid (arrow) and gallbladder wall thickening. Calculi are shown in the proximal common bile duct (CBD, curved arrow) consistent with choledocholithiasis. (b) Fluoroscopic image from the same patient during endoscopic retrograde cholangiopancreatogram (ERCP) and sphincterotomy. The dilated CBD is outlined by iodinated contrast with guide wire in situ. Filling defects in the distal CBD consistent with calculi (arrow).

Cholelithiasis may arise due to increased faecal bile acid secretion secondary to pancreatic insufficiency which results in lithogenic bile [28]. Cholangiopathy occurs in the form of intrahepatic bile duct dilation (Figure 1(c)), beading, or stricture formation and has been seen in children and adults with CFLD. Bile duct abnormalities have also been reported in a significant number of CF patients without clinically apparent liver disease [29]. Imaging findings include intra- and extrahepatic biliary strictures (Figures 4(a) and 4(b)), cholelithiasis, and microgallbladder (Figure 5(a)). Choledocholithiasis and cholecystitis are also reported (Figure 6).

### 4.4. Hepatic Steatosis

**Figure 7:**
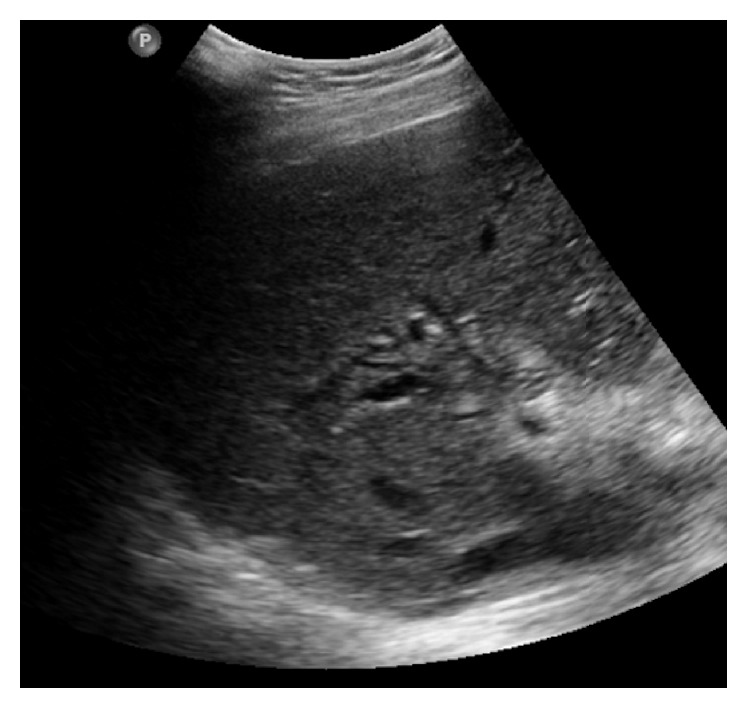
Longitudinal US of an asymptomatic 21-year-old male with cystic fibrosis performed for screening purposes. Echogenic liver parenchyma is consistent with hepatic steatosis.

Steatosis is one of the most common hepatic manifestations in the CF population, occurring in 23–75% of CF patients [30]. Patients may present with smooth hepatomegaly but are usually asymptomatic, without signs of portal hypertension. It is represented by increased liver echogenicity on US (Figure 7), low attenuation on CT, and decreased T2 signal intensity on MRI.

Steatosis was previously accepted as a benign entity in both CF [31] and the general population [32]. However, the rising prevalence of nonalcoholic fatty liver disease (NAFLD) has brought increased focus on steatosis, and while nonalcoholic steatohepatitis (NASH) was traditionally considered the only form of NAFLD to confer considerable risk of fibrosis progression, recent studies have now drawn attention to a previously underappreciated risk of fibrosis progression in patients with steatosis [33, 34].

In a meta-analysis including 11 studies, of which 133 patients had NAFLD with paired biopsies, 39.1% developed progressive fibrosis. The annual fibrosis progression rate in NAFLD patients with stage 0 fibrosis at baseline was 0.07 stages compared with 0.14 stages for NASH patients. Pais et al. observed that 13 of 16 patients with NAFLD and mild inflammation at baseline progressed to typical NASH or bridging fibrosis, while only 1 of 5 patients with simple steatosis progressed to NASH [34]. Overall, these studies suggest that NAFLD patients can develop progressive fibrosis though at a slower rate than those with NASH and cirrhosis may develop if steatosis progresses to steatohepatitis and subsequent fibrosis. As our understanding of the natural history of steatosis in NAFLD evolves, our understanding of its significance within the CF population may also evolve.

### 4.5. Fibrosis: Focal Biliary Cirrhosis

Focal biliary cirrhosis (FBC), characterised by focal portal fibrosis and cholestasis, is a histopathologic lesion that is typical of CF related liver disease. It arises as a result of biliary obstruction and progressive periportal fibrosis and can progress to multilobular cirrhosis and portal hypertension, albeit very rarely [20]. It is typically clinically silent without raised transaminases.

**Figure 8:**
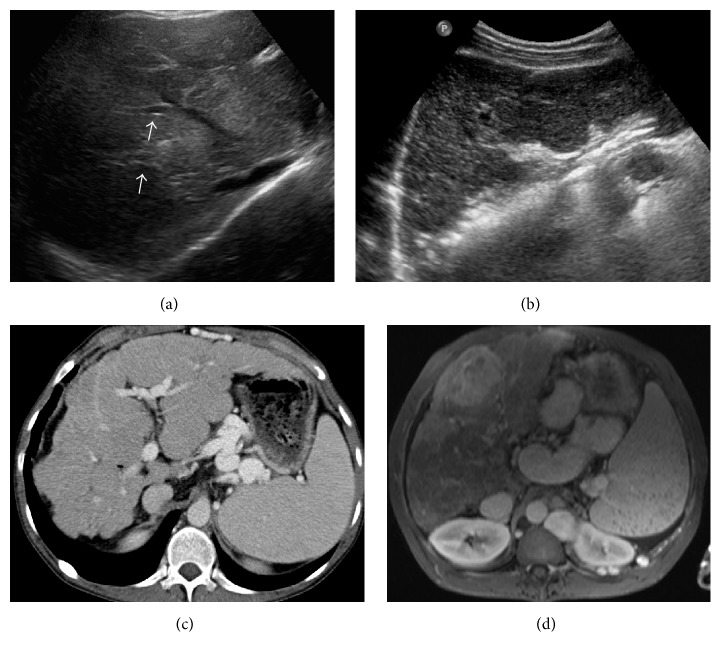
(a) Longitudinal US image of a 32-year-old male with cystic fibrosis demonstrating periportal echogenicity (arrows) consistent with periportal fibrosis. (b and c) Transverse US image and axial CT demonstrating a shrunken, nodular right lobe of liver with relative left lobe hypertrophy, varices, and splenomegaly in a CF patient with deranged LFTs at time of imaging. (d) Delayed enhanced T1-weighted axial MRI demonstrating regenerative nodules surrounded by bands of enhancing fibrotic liver parenchyma.

The typical US feature is periportal echogenicity (Figures 8(a) and 8(b)). This correlates with pathologic findings of cells containing fat globules in the fibrotic portal tracts and cells in the periportal liver parenchyma which contain large fat-laden vacuoles [27]. MRI findings include high intensity signal in periportal areas on T1 weighted imaging (Figures 8(c) and 8(d)) [7].

### 4.6. Multilobular Cirrhosis

Although rare, multilobular cirrhosis is the most clinically significant form of CFLD and has an estimated average prevalence of 5.6% as per one review of 12 reports including 4446 patients [19]. A prospective study of 177 patients demonstrated a median age at diagnosis of 7 years (range; 2 months–18 years) with an incidence rate of 2.5 per 100 patient years for the first decade of life which sharply declines thereafter [35]. Multilobular cirrhosis carries with it the risk of portal hypertension and varices with a prevalence of 4.2% and 2.4%, respectively [19].

Liver disease in CF is often silent until late stages of the disease. Even with the development of multilobular cirrhosis, patients can remain asymptomatic until signs of portal hypertension develop [19]. Clinically, multilobular cirrhosis presents as a hard nodular liver with or without hepatomegaly.

Pathologically, it is characterised by diffuse cirrhosis with multiple regenerative nodules and imaging reveals a nodular liver contour with coarse heterogeneous parenchyma (Figure 8(b)) [6]. On CT and MRI, multiple regenerative nodules are seen, surrounded by bands of T2 hypointense fibrosis (Figures 8(c) and 8(d)) [27].

### 4.7. Complications in CF Cirrhosis 

#### 4.7.1. Portal Hypertension and Related Complications

**Figure 9:**
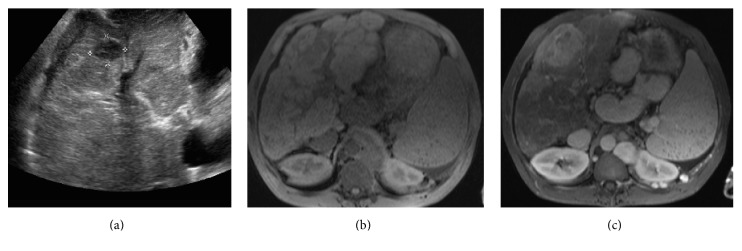
(a) Longitudinal US image of a 34-year-old male with cystic fibrosis was done as part of routine surveillance for cirrhosis in the context of stable biochemical markers. US image demonstrating a well-circumscribed hypoechoic lesion. (b and c) Noncontrast and arterial phase T1-weighted gadolinium enhanced MRI demonstrating a hyperenhancing well-circumscribed lesion in segment 4. Splenic siderotic nodules are an additional feature of portal hypertension.

If portal hypertension complicates the presentation of multilobular cirrhosis, patients may present with varices, ascites, or splenomegaly which can be readily identified on US and CT (Figures 8(b) and 8(c)). Siderotic nodules in the spleen, also known as Gamna-Gandy bodies, are another feature of portal hypertension seen on MRI and are due to haemorrhage within the spleen (Figure 9) [27]. Of note, portal hypertension can present in noncirrhotic patients [19].

Rowland and colleagues have identified CFLD with portal hypertension as an independent risk factor for mortality, with a 10-year pair-matched cohort demonstrating an almost threefold increased risk of death compared to CF controls without liver disease [5]. However, the literature regarding this finding suggests that this is not a uniformly held opinion [35–37].

#### 4.7.2. Hepatocellular Carcinoma

HCC can occur in patients with cirrhosis of any aetiology. With increased survival in patients with CF, there is a risk of HCC. Four such cases have been reported in the literature [6, 15–17]. Typical imaging features of this lesion in a 35-year-old male with CF and cirrhosis are depicted in Figure 9. HCC is usually characterised by a well-circumscribed lesion which is hypoechoic on US (Figure 9(a)) and low signal intensity on unenhanced T1-weighted MRI (Figure 9(b)) with avid enhancement on arterial phase MRI using gadolinium contrast (Figure 9(c)) [38]. Contrast washout on delayed phase imaging may also be seen. Similar enhancement features are seen using dynamic enhanced CT.

The American Association for the Study of Liver Disease (AASLD) guideline update in 2010 recommends biannual US for screening of HCC in any patient with cirrhosis, with reliance upon imaging and/or biopsy for diagnosis. Nodules greater than 1 cm should be assessed with 4-phase multidetector CT or dynamic contrast-enhanced MRI to assess for arterial hyperenhancement and portal venous or later phase washout. If these findings are absent, the recommendation is to proceed to further contrast-enhanced imaging or biopsy [39].

### 4.8. Imaging and the Challenges of Early Diagnosis of CFLD

Although CFLD is the most significant abdominal manifestation of CF from a mortality perspective, it remains the most challenging in terms of early recognition and diagnosis. We therefore dedicate this section to discussing the current challenges and future perspectives on the diagnosis of CFLD as it pertains to imaging.

Ultrasound is more sensitive than LFTs in detecting CFLD [20], with suggestions that abnormal echogenicity can precede clinical or biochemical manifestations of liver disease. However, US has been shown to have a positive predictive value of only 33% and while an abnormal US may predict the presence of moderate to severe liver disease, a normal US does not exclude significant liver fibrosis [40].

Biopsy is reserved for cases of diagnostic doubt which would change management [20]. However, some studies suggest histopathological confirmation and staging of fibrosis by liver biopsy at initial diagnosis of CFLD may be warranted given its superior performance in predicting clinically significant CFLD and portal hypertension compared to noninvasive alternatives currently used in screening and follow-up [21]. In a 12-year prospective analysis of 40 children with CF, Lewindon and colleagues demonstrate that dual-pass percutaneous biopsy decreases sampling error and can predict portal hypertension while abnormal clinical exam, liver function tests (LFTs), and US failed to predict either presence of fibrosis or occurrence of portal hypertension [21].

Currently, however, best practice guidelines advise screening for CFLD using basic laboratory markers (LFTs, platelets, and INR) and abdominal US. Further imaging with CT or MRI is recommended if liver lesions or biliary tract involvement is found on US without sufficient clarity for diagnosis [20].

CT and MRI are useful in distinguishing fibrosis from steatosis, which can be difficult to determine on US, and are also useful for further investigation of focal lesions [20]. The advantage of MRI is that it is a comprehensive noninvasive investigation of the liver, biliary tract, and pancreas without ionising radiation exposure, which is an important consideration given the need for possible follow-up. Durieu et al. demonstrated the capacity of MR to detect intrahepatic biliary abnormalities in CF patients without clinically apparent liver disease, pointing to a likely underestimation by US and to MRCP as an option to identify early biliary abnormalities [29].

Transient elastography (TE) measures liver stiffness and is a mechanism for staging fibrosis which is already validated for chronic liver conditions such as hepatitis C. Early studies using TE for noninvasive assessment of liver fibrosis in CF patients showed no clear improvement over US [41]. ARFI is a novel US based elastography-method which, unlike fibroscan, can be used on both lobes of the liver [42]. Later studies proposed both fibroscan and ARFI as sensitive diagnostic tools for fibrosis [42] and provided some evidence for its use as a reliable detection tool for CF cirrhosis [43] with potential in screening for portal hypertension [44]. One prospective study using ARFI and TE supported feasibility of these tests in assessment of liver fibrosis in CF; however, further studies with longer follow-up are required [45].

A consensus on cut-off values for early diagnosis of CFLD remains elusive, and further research in this area is required, although this may in part be attributed to the absence of an accepted consensus definition of CFLD [45]. Ultimately, TE shows promise and, with further study, may improve diagnosis of liver fibrosis in CF patients if used in conjunction with current screening methods. While there are no biopsy-controlled studies for this technique in CFLD, authors cite the established evidence for TE's correlation with fibrosis in other liver diseases and have questioned the ethics of biopsy-controlled studies in paediatric populations due to its invasive nature, associated risks, and issues with respect to sampling error [41].

## 5. Conclusion

The abdominal manifestations of CF are of increasing importance in light of increased survival of adult populations with CF. Early recognition and diagnosis of these conditions and their complications will play an important role in improving the quality of life and further survival of patients with CF. Significant CFLD with cirrhosis or portal hypertension poses one of the biggest challenges in this respect.

Abnormal clinical examination, LFTs, and US findings are poor predictors of progression to cirrhosis or portal hypertension [21]. Investigations that reliably predict cirrhosis or portal hypertension are needed to better identify those at risk and to instigate earlier preventative measures and management of complications. A number of promising techniques may ultimately contribute to achieving this goal, but further research is awaited.

An awareness of the abdominal manifestations of CF and familiarity with their expected clinical presentation and imaging findings are paramount to the timely diagnosis and appropriate management of patients with CF.

## Figures and Tables

**Table 1:** Proposed classification of CFLD by North American CF Foundation (2007) adapted from Flass and Narkewicz [19].

(1) Preclinical: No evidence of liver disease on exam, imaging, or laboratory values
(2) Liver involvement without cirrhosis/portal hypertension: at least one of
Biochemical abnormalities
(a) Persistent AST, ALT, and GGT >2 times upper limit of normal
(b) Intermittent elevations of the above laboratory values
Cholangiopathy (based on US, MRI, CT, and ERCP)
Steatosis
Fibrosis
US abnormalities not consistent with cirrhosis
(3) CF related liver disease with cirrhosis/portal hypertension

AST: aspartate transaminase, ALT: alanine transaminase, and GGT: gamma-glutamyl transferase.
